# A Generalized Allosteric Mechanism for *cis*-Regulated Cyclic Nucleotide Binding Domains

**DOI:** 10.1371/journal.pcbi.1000056

**Published:** 2008-04-11

**Authors:** Alexandr P. Kornev, Susan S. Taylor, Lynn F. Ten Eyck

**Affiliations:** 1 San Diego Supercomputer Center, University of California San Diego, La Jolla, California, United States of America; 2 Department of Chemistry and Biochemistry, University of California San Diego, La Jolla, California, United States of America; 3 Howard Hughes Medical Institute, University of California San Diego, La Jolla, California, United States of America; HHMI/UT Southwestern Medical Center, United States of America

## Abstract

Cyclic nucleotides (cAMP and cGMP) regulate multiple intracellular processes and are thus of a great general interest for molecular and structural biologists. To study the allosteric mechanism of different cyclic nucleotide binding (CNB) domains, we compared cAMP-bound and cAMP-free structures (PKA, Epac, and two ionic channels) using a new bioinformatics method: local spatial pattern alignment. Our analysis highlights four major conserved structural motifs: 1) the phosphate binding cassette (PBC), which binds the cAMP ribose-phosphate, 2) the “hinge,” a flexible helix, which contacts the PBC, 3) the β_2,3_ loop, which provides precise positioning of an invariant arginine from the PBC, and 4) a conserved structural element consisting of an N-terminal helix, an eight residue loop and the A-helix (N3A-motif). The PBC and the hinge were included in the previously reported allosteric model, whereas the definition of the β_2,3_ loop and the N3A-motif as conserved elements is novel. The N3A-motif is found in all *cis*-regulated CNB domains, and we present a model for an allosteric mechanism in these domains. Catabolite gene activator protein (CAP) represents a *trans*-regulated CNB domain family: it does not contain the N3A-motif, and its long range allosteric interactions are substantially different from the *cis*-regulated CNB domains.

## Author Summary

Cyclic nucleotides are small regulatory molecules which transmit signal from receptors positioned on a living cell membrane into the cell interior and regulate multiple biological processes ranging from bacteria to humans. Such regulation occurs through binding of the cyclic nucleotides to the corresponding proteins. All such proteins contain a relatively small domain responsible for the cyclic nucleotide binding. The most important task is to understand how cyclic nucleotide binding (CNB) domains translate the signal into a biological response. In this work, we studied changes in different CNB domains induced by the cyclic nucleotides using a new method for comparison of protein structures: local spatial patterns alignment. This novel method compares protein molecules and detects conserved spatial patterns comprised of similar amino acid residues. Moreover, it ranks the detected residues with respect to their functional or structural importance. Our results show that there are at least two different families of CNB domains. The first family has four structural elements which perform the signal translation. Two of these elements were known previously and two are novel. The second family can translate the signal using only three elements, but they have to work in pairs to provide interaction between the functional elements.

## Introduction

**Figure 1:**
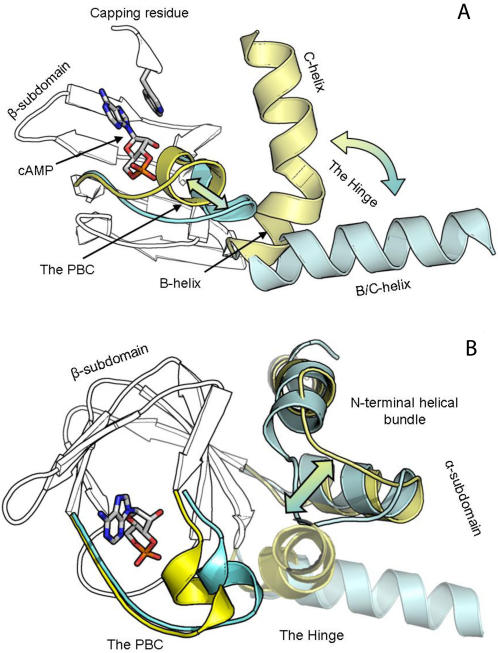
Current understanding of the major cAMP–CNB domain interactions represented by the PKA regulatory subunit type Iα: A-domain. (A) Correlated movement of the PBC and the Hinge. cAMP-bound conformation (B-form) is colored yellow; holoenzyme conformation (H-form) is colored cyan. The cAMP and the capping residue positions are shown. (B) N-terminal part of the α-subdomain performs reverse motion (with respect to the Hinge), contacting the PBC in the H-form.

Cyclic adenosine monophosphate (cAMP) is an important second messenger, which regulates a large variety of cellular processes, including metabolism, cell shape transformation, gene transcription, photoreception and chemosensation [1]–[5]. All cAMP-binding proteins in both pro- and eukaryotes share a small module – the cyclic nucleotide binding domain (CNB domain), which is typically fused to another domain. The CNB domain contains a contiguous β-subdomain and a non-contiguous α-subdomain (Figure 1). The former is a relatively rigid eight-stranded β-sandwich, which accommodates the cyclic nucleotide molecule. The flexible helical α-subdomain can accept substantially different configurations, which translates the allosteric signal [6]. Recent structure studies of cAMP-dependent protein kinase (PKA) demonstrated, that CNB domains toggle between two stable conformations: bound to cAMP (so called B-form [7]), or to catalytic subunit of PKA (H-form) [8],[9]. The intermediate, non bound form (apo-form) is characterized by high backbone flexibility [10]–[12] and is apparently represented by a dynamic ensemble of multiple configurations.

The β-subdomain contains a highly conserved element, the Phosphate Binding Cassette (PBC), which is 14-residues long and contains a short flexible α-helix (B'-helix). Ribose-phosphate of cAMP, protected by the β-sandwich from the outside solution, forms six strong hydrogen bonds to the PBC. Due to these interactions, the B'-helix moves towards the cAMP molecule and adopts a compact conformation (Figure 1). Such movement causes a substantial rearrangement of the α-subdomain both in its N- and C-terminal parts. The latter contains the so called “hinge” [13], which consists of two consecutive α-helices (B and C). These helices are remarkably flexible and due to a strong connection to the B'-helix perform a swing-like motion: moving towards cAMP in the B-form (Figure 1A). The N-terminal part of the α-subdomain, which typically consists of two (short) helices and was called the “N-terminal helical bundle” [14], moves in an opposite way; in the B-form it moves away from the PBC, facilitating the hinge closure. In the H-form the N-terminal helices move towards the cAMP and make a contact to the B'-helix, filling the void space that results from the hinge opening (Figure 1B).

The other part of the cAMP molecule, the adenine ring, acts as a hydrophobic moiety, which stacks against a “capping residue” in all known CNB domain structures [15] (Figure 1A). Mutation studies have established the importance of this contact for stabilization of the B-form and cooperative cAMP-induced activation of the PKA holoenzyme [8].

A recent review summarizes this information into a general model for the CNB domain allosteric mechanism for PKA, Rap guanine nucleotide-exchange factor (Epac) and hyperpolarization-activated cyclic-nucleotide-modulated channel (HCN) [14]. Although this model is in a good correspondence with much experimental data, two important issues remained unclear. First, as the authors mentioned, the N-terminal helical bundle is replaced by a single helix in the catabolite gene activator protein (CAP). This raises a question about the role of the helical bundle and its functional and structural conservation. Is it a universal part of the CNB domain or it is a part of protein-protein interface between the CNB domain and the host protein? The second problem is related to the loop located between β_2_ and β_3_ strands. A series of publications demonstrated, that it is an important element of allosteric mechanism in the PKA RIα∶A-domain [16]–[18], but it was not included in the model described by Rehmann et al [14] and was not considered to be a universal element.

To elucidate the cAMP induced allosteric mechanism that is conserved in different CNB domains, we used a recently developed method for protein structure comparison: Local Spatial Patterns (LSP) alignment that is capable of detecting similar patterns made up by amino acid residues in space. It is fast and does not require preliminary sequence or structural alignment of the compared proteins. Earlier we used it for comparison of protein surfaces of several CNB domains in the B-form and detected a conserved set of hydrophobic residues protecting the cAMP ribose-phosphate [15]. Here we considered both water accessible and buried residues of both B- and H-forms of four different CNB domains: PKA, HCN, Epac and bacterial cyclic nucleotide modulated potassium channel (MloK1) [19]. The recently reported structures of two PKA holoenzymes [8],[20] have allowed us for the first time to analyze both conformational states of multiple CNB domains.

Our analysis has shown that there are four elements conserved in all known CNB domains, with the exception of the CAP: the PBC, the hinge, the β_2,3_-loop and the “N3A-motif”. The latter consists of the A-helix, a preceding eight residue loop and a short N-terminal helix. The loop contains a set of 3_10_-turns, and is termed “the 3_10_-loop”. Based on these results, we propose a general model for the allosteric mechanism in CNB domains, which we called *cis*-regulated domains. In CAP the N3A-motif is reduced to a single A-helix. The difference between CAP and other CNB domains is discussed.

## Results

### LSP Alignment Defines cAMP-Induced Conformational Changes in CNB Domains

The LSP alignment is a new method to compare protein molecules. It is based on a graph-theoretical representation of protein structure, and the result of this alignment is a pair of isomorphic graphs. Vertices of the graphs correspond to the residues which form similar spatial patterns in both proteins. Each vertex/residue is connected to the rest of the graph by several edges. They indicate the residue neighbors whose positions and orientation in space are conserved with respect to this residue. As we have shown earlier [15],[21], functionally important residues of protein kinases have numerous connections on the similarity graphs. In the previous works we considered only surface exposed residues. Here we analyze all residues. This allows us to recognize conserved motifs that are buried in the protein core. We define a term “involvement score” (IS) of a particular residue, which is equal to the number of edges for the corresponding vertex on the graph provided by the LSP alignment procedure. It reflects the extent of participation of this residue in formation of invariant spatial patterns and corresponds to AA and AI scores used in the previous work [21], where we compared active and inactive protein kinases.

Earlier we used the LSP alignment for comparison of different proteins having similar functions. In this work we present an alternative way of using the LSP alignment program. Our purpose was to quantify cAMP-induced structural rearrangements in different CNB domains. This was made by aligning two different conformations of the same protein. As the IS reflects only local structural similarities (in our case within 10 Å range between C_α_-atoms) any large scale rearrangements in the protein do not change the score significantly. Therefore, residues which form rigid structures inside the protein and maintain their relative positions will have a high level of IS. In contrast, those residues located in points of protein flexibility will have low IS, reflecting the loss of similarity between the two protein structures. One can speculate that the residues with the lowest IS can play an important role in the allosteric mechanism, as such elements like “hinges” or “switches” have to accept two distinctive “on” and “off” conformations.

### Recognition of a Conserved Novel Allosteric “N3A-Motif”

**Figure 2:**
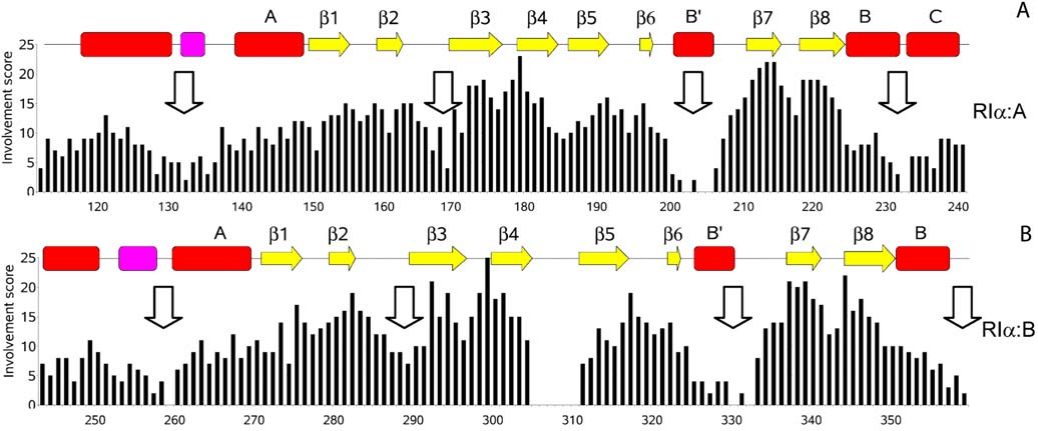
Study of the cAMP-induced conformational changes in PKA∶RIα by LSP-alignment. High involvement scores correspond to relatively rigid parts of the molecule. Low values of the score characterize elements, which are the most sensitive to the presence of cAMP. Secondary structure is shown by red rectangles (α-helix), magenta rectangles (3_10_-helix), and yellow arrows (β-strands). Four allosteric “hot spots” for each domain are shown by arrows.

Figure 2 presents the results of LSP alignment of PKA (RIα) H- and B-forms. As expected, residues from the rigid β-sandwich had the highest IS values, reflecting the rigidity of the β subdomain. In contrast, four regions showed a significant decrease in IS: 1) B'-helix of the PBC; 2) the hinge region; 3) the β_2,3_-loop and 4) N-terminal part of the α-subdomain. These results are in good correspondence with the NMR studies of the RIα A-domain [16]. Our analyses show that both A- and B-domains have similar IS profiles, although the drop in the β_2,3_-loop in B-domain was less prominent. Similar results were obtained for the A-domain of PKA (RIIβ) and the potassium channel MloK1 (Figure S1). The decrease of IS in the β_2,3_-loop in the potassium channel CNB domain was not as striking as in A-domains of PKA (both RIα and RIIβ), but similar to the B-domain of RIα. This is in a good agreement with the earlier observation that B-domains of PKA are more similar to the rest of CNB domains, than the A-domains [15].

**Figure 3:**
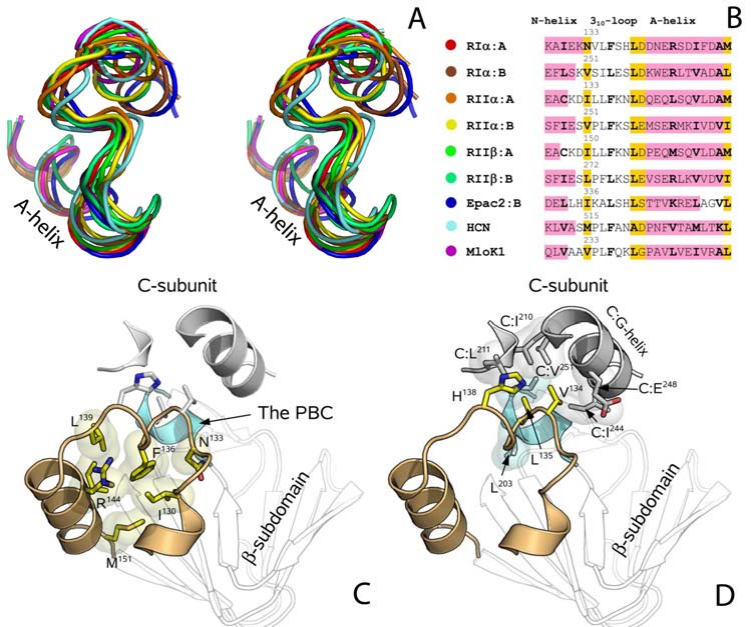
The conserved N3A-motif in the N-terminal part of the α-subdomain. (A) Stereo picture of N3A-motifs from 9 different CNB domains: A and B domains of PKA types RIα, RIIα, and RIIβ; Epac2; ionic channel HCN; and potassium channel MloK1. (B) Sequence alignment of the 9 N3A-motifs. α-helical regions are shaded magenta. Residues with negative chirality are shaded yellow. Hydrophobic residues or residues with large aliphatic segments are shown in bold. Colored circles correspond to the coloring on the stereo picture. (C) Hydrophobic interactions between residues of the N3A-motif provide integrity of the structural element. N3A-motif of PKA∶RIα A-domain is colored tan. Interacting residues are colored yellow. Connelly surfaces around their aliphatic parts are shown. (D) Residues on the tip of the 3_10_-loop are involved in protein-protein interactions in the PKA holoenzyme. The PBC is colored cyan. C-subunit is colored grey.

The N-terminal helical structure which earlier was called an “N-terminal helical bundle” has not been considered as a conserved element [14]. It is not a part of the current CNB domain nomenclature for two reasons: first – it is not present in CAP, and second – in the different CNB domains it forms slightly different secondary structures. For example in the B-form of RIα, RIIβ, MloK1 and Epac2 it contains a short 3_10_-helix. In the H-form of RIα∶A it contains a set of 3-turns, which do not form the classical 3_10_-helix and is considered to be a loop, while the B-domain retains its 3_10_-helix configuration. In H-form of PKA RIIα∶A and RIIβ∶A this element qualifies as an α-helix, but in the B-form of RIIβ∶A and RIIβ∶B it is a 3_10_-helix. However, a close look at the middle part of the bundle shows that the geometry of its backbone in different CNB domains (both H- and B-forms), is rather conserved (Figure 3A). Moreover, this element, which we will define as a “3_10_-loop”, has a distinctive pattern of phi/psi angles: Figure 3B shows a sequence alignment of different N-terminal helical bundles. One can see that A-helix of the presented CNB domains is preceded by an eight residue long loop. Its first conserved feature is that both ends of the loop contain residues with negative chirality (a characteristic of β-strands): one at the N-terminus and two at the C-terminus. There is also a large conserved hydrophobic residue (phenylalanine or leucine) in the middle of the loop (F^136^ Figure 3C), which plays a central role in the hydrophobic cluster formed by the A-helix and the preceding α-helix, which did not have an established name. As in the PKA-RIα A-domain, it was called “αX_n_-helix” [16] or “X∶N-helix” [6], here we call it “N-helix”, and the combination of the N-helix, the 3_10_-loop and A-helix structure – the N3A-motif. The characteristic feature of this motif is the presence of multiple X–X pairs in its sequence (where X represents a hydrophobic residue or a residue with large hydrophobic segment such as arginine or asparagine) (Figure 3B). Such residues are closely positioned on one side of the helix and provide a secure connection between the N3A-motif elements (Figure 3C), a feature similar to the tetratricopeptide repeat [22] or the leucine-zipper [23] motifs.

Analysis of the recently discovered holoenzyme structures of PKA shows that the 3_10_-loop residues positioned between the X–X pairs are usually involved in important interactions. For example, in RIa∶A-domain V^134^, L^135^ and H^138^ stack against a large hydrophobic cluster formed by the C-subunit and the PBC (Figure 3D). In the RIa∶B-domain S^252^ forms five hydrogen bonds to the PBC, the C-terminal helical structure of the domain and the β-sandwich.

The suggested conservation of the N3A-motif in different CNB domains raises a question about the definition of A and B domains in PKA-R. Until now, the beginning of the B domain was associated with the first residue in it's a-helix (e.g. W^260^ in RIα or V^280^ in RIIβ). Here we suggest a new boundary between the A and B domains, which will reflect the conservation of the N3A-motif. It is known that the RIα-(94-244) construct retains its functionality and is capable of both binding to cAMP and regulating PKA [24]. In addition, the C-helix of cAMP-bound RIα has a kink between Y^244^ and E^245^. An identical kink exists in the cAMP-bound RIIβ (between Y^265^ and E^266^). It seems logical to suggest that this kink indicates the border between the A and B domains, therefore defining E^245^ as the beginning of N3A-motif for the B-domain of RIα. Such a definition supports the observation made earlier by Huang and Taylor that RIα “residues 245–260 at the end of cAMP binding domain A are structurally more a part of domain B than domain A” [24].

### LSP Alignment of Different CNB Domains Emphasizes the Allosteric Role of the β_2,3_-Loop

**Figure 4:**
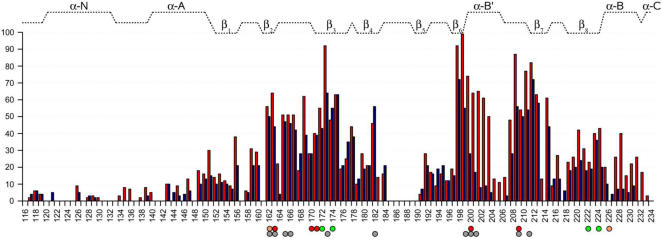
Accumulated involvement scores obtained by LSP alignment different CNB domains. B- form of RIα∶A was compared to B-forms of RIα∶B, RIIβ∶A, RIIβ∶B, HCN, and MloK1 (red bars); and H-forms of RIα∶B, RIIα∶A, RIIα∶B, Epac, and MloK1 (blue bars). Colored circles indicate the “three-shell” model[16] residues: 1^st^ shell, red; 2^nd^ shell, yellow; and 3^rd^ shell, green. Dark grey circles indicate the residues, which were found to be conserved previously [15].

After the new definition of CNB domains, we used the LSP alignment to detect residues involved in formation of conserved spatial patterns as we did previously for PKA-C [21]. The A-domain of the RIα holoenzyme was taken as a reference structure. It was compared to five cAMP-bound CNB domains: RIα∶B, RIIβ∶A, RIIβ∶B, HCN and MloK1. To detect the regions, which respond to the cAMP presence, we also compared our reference structure to five cAMP-free CNB domains: six domains of PKA-R taken from the corresponding holoenzyme complexes: RIα∶B, RIIα∶A, RIIα∶B; and two apo-structures: Epac and MloK1. Involvement scores were accumulated and presented in Figure 4.

The highest involvement scores were detected in the PBC and neighboring β_6_ and β_7_ strands. This area also had the largest cAMP-induced changes of IS, reflecting the leading role of the PBC in the allosteric mechanism. Reduced scores in the middle of PBC agree with the sequence variability profiles obtained earlier for PKA-R [25].

**Figure 5:**
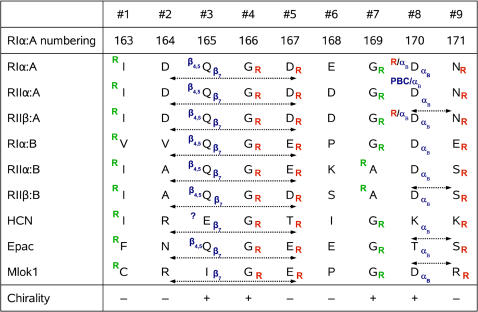
Sequence alignment of β_2,3_-loops for different CNB domains. Contacts formed by the residues are shown either on the upper left side (side chain) or lower right side (main chain). Contacts to the R^209^ are indicated by capital R: green, hydrophobic; red, polar. Also contacts to the PBC, B-helix, β_4,5_-loop, and β_7_-strand are indicated. Question mark signifies that the β_4,5_-loop is not resolved in the HCN structure. Dashed arrows show important hydrogen bonds: above the residue letters, between side chains; under the letters, between their main chains. The last row presents main chain chirality sign for the residues.

**Figure 6:**
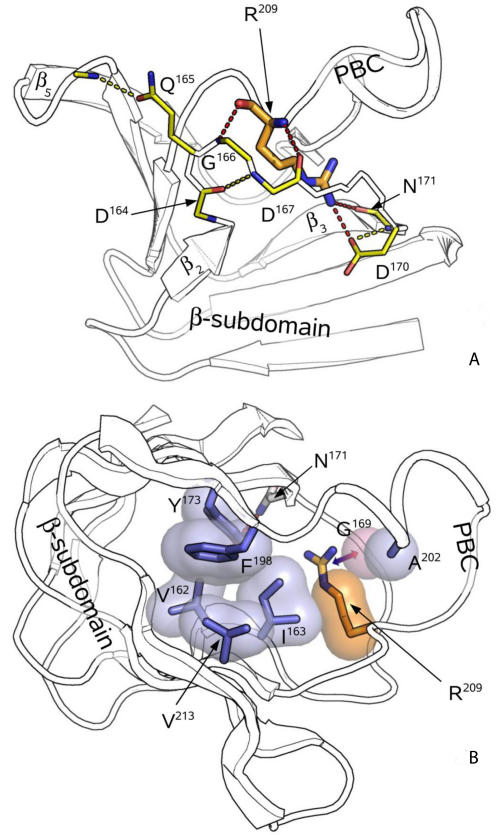
Highly conserved β_2–3_-loop secures the PBC-arginine side chain position. (A) Major polar interactions of the β_2–3_-loop (RIα∶A case). R^209^ is colored tan. Other residues involved in the interactions are colored yellow. (B) Nonpolar polar interactions surrounding the PBC-arginine. Hydrophobic residues are colored blue, and their Connelly surfaces are shown. CH-π interaction between the conserved G^169^ and R^209^ is indicated by an arrow.

The second major region with highly scored residues was in the strands β_2_, β_3_ and the loop between them. The result was rather unexpected as, until recently, this area was not considered as an important part of CNB domains. The scores, in general, did not depend on the presence of cAMP, which indicates overall conservation of the loop geometry. This suggests, that the β_2,3_-loop is an important element not only in PKA-R, as it was pointed by Das et. al [16],[26] , but in all CNB domains. Figure 5 shows a comparison between β_2,3_-loops of different CNB domains. As was detected by the DSSP program [27], all loops have the same pattern of their main chain chirality, indicating a high level of their geometry conservation. The most distinctive common feature for all of them is a 3-turn between residues #2 (D^164^) and #5 (D^167^) (Figure 6A). It contains an invariant glycine residue #4 (G^166^) which makes a conserved hydrogen bond to the PBC-arginine carbonyl. The reason for strict conservation of the glycine is evident as its dihedral angles are ruled out for any other type of residue (*φ* = 89.4° and ψ = −26.8° for RIα∶A). Another conserved hydrogen bond is formed between the PBC-arginine amide and carbonyl of the residue #5. The third polar contact, which can be found in all CNB structures, is formed between the PBC-arginine guanidinium group and the carbonyl of residue #9 (N^171^). The hydrogen bond between the arginine and the side chain of residue #8 (D^170^), which was found to be important for RIα∶A, is not conserved: it can be seen only in the A domains of RIα and RIIβ. This residue, however, often binds to the side chain of residue #9 and provides communication between the β_2,3_-loop and the “hinge” as its carbonyl is always bound to the amide group of the first residue in the B-helix. Besides polar interactions with the PBC-arginine, residue #1 (I^163^) makes a conserved hydrophobic contact to the arginine side chain. This residue is a member of a conserved hydrophobic core formed by the highly scored residues: V^162^, I^163^, Y^173^, F^198^ and V^213^ (Figure 6B). The important detail is that another member of that cluster: Y^173^ (one of the three highest scores obtained) – makes a conserved hydrogen bond to the residue #9 (N^171^), thus closing the circle around the PBC arginine.

Glycine is the predominant residue in position #7, except for the B domains of RIIα and RIIβ, where it is substituted by alanine. Any increase of the side chain would lead to a steric clash with highly conserved alanine residues from the PBC (A^202^), which in its turn also has a hydrophobic contact to the PBC-arginine in the cyclic nucleotide bound configurations.

The hinge, which is a well known element of the allosteric mechanism, demonstrated medium levels of IS and a strong dependence on the presence of cAMP. The preceding β_8_-strand received almost the same level of scores. It contains a set of conserved hydrophobic residues, which face the N3A-motif and provide a secure connection of this element to the β-sandwich. The NMR-study of RIα showed that two residues from the β_8_-strand (W^222^ and I^224^) had a substantial chemical shift, in response to cAMP binding and are a part of the allosteric mechanism. Our results support this conclusion and demonstrate conservation of the hydrophobic interface through different CNB domains. Another highly scored residue positioned between B-helix and β_8_-strand (M^151^) is also a part of this interface.

As we showed earlier the N3A-motifs of different CNB domains have very similar geometry, and conserved sequence motifs. However, this region, except for the C-terminus of A-helix, demonstrated low levels of IS. This indicates that the residues, conserved in the sequence, do not form a rigid spatial motif. This conclusion is supported by the fact that the N-helix and 3_10_-loop in RIα∶A-domain have an elevated level of hydrogen-deuterium exchange [10],[16]. Apparently, the N3A-motif is a rather flexible element, which can adopt slightly different conformations accommodating the PBC and the hinge movements.

In all comparisons the loop between β_4_ and β_5_-strands received zero level of IS (Figure 4). It is consistent with the fact that this part of the CNB domain is the least conserved in terms of sequence and structure [28]. Our data show that in many CNB domains the N-terminus of the β_4–5_-loop involved in the conserved anchoring of the β_2–3_-loop via its #3 residue (Q^165^) (Figures 5 and 6A). The β_4–5_-loop spatially comes close to the C-terminus of the CNB domain and in the RIα∶B-domain may provide a docking site for another protein.

### CNB Domains in CAP Are *trans*-Regulated

**Figure 7:**
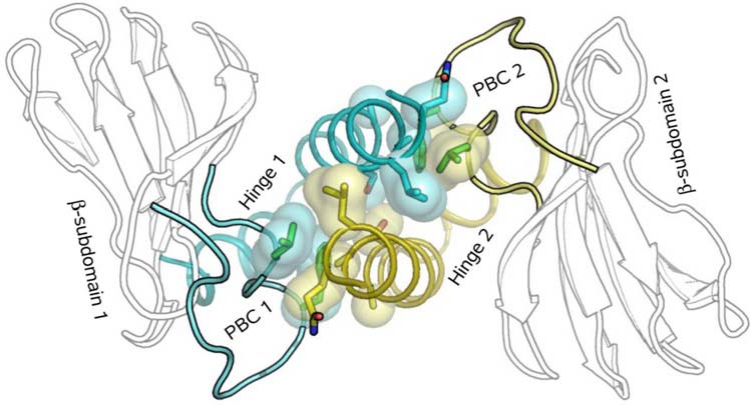
CNB domains of CAP are *trans*-regulated. Interface between two monomers of CAP is shown (PDB ID, 1CGP). β-subdomains are shown as black and white contours. The PBC and the Hinge of each monomer are shown as cartoons and colored cyan (first monomer) and yellow (second monomer). Residues which form the hydrophobic interface between PBCs and hinges are shown as sticks with Connelly surfaces.

We deliberately excluded the CAP CNB domain from the current analysis as it does not contain the classic N3A-motif. There are several distinctive features that distinguish the CAP CNB domain from those discussed above. The major difference is that in functionally active CAP the two identical CNB domains form a homodimer with the interface being formed mainly by their C-helices. Regulatory subunits of PKA also contain two CNB domains, but their mutual interaction is rather limited: e.g. A-helix of B-domain contacts cAMP and the hinge from the A-domain. In contrast, in CAP the interaction between the two monomers is the most important allosteric contact between the PBC and the hinge. Figure 7 shows that the major binding partner for each PBC is not the hinge from its own CNB domain but the hinge from the opposite monomer. Such interactions separate the CAP CNB domain from the other CNB domains studied in this work, which we propose to call *cis*-regulated CNB domains while defining CAP CNB domain as a *trans*-regulated CNB domain.

## Discussion

The CNB domain, highly conserved throughout biology, is comprised of a set of motifs that both define the docking site for cAMP as well as an allosteric switch that allows it to assume a dramatically different structure when binding to another protein element. Using our new computational method we have analyzed both the allosteric mechanism and the structural motifs conserved in different CNB domains. LSP-alignment proved to be an effective tool for detection of the allosteric “hot spots” – residues with the most significant rearrangement of their side chains. The comparison of H- and B-forms of PKA-RIα pointed to four major elements of the allosteric mechanism: the PBC, the hinge, the β_2,3_-loop and the N-terminal helical structure. Based on our analysis the latter is now recognized as a new conserved element of *cis*-regulated CNB domains – the N3A-motif. It is not present in at least one *trans*-regulated CNB domain – CAP; however, it can be found in another transcriptional regulator, Crp/Fnr family from Porphyromonas gingivalis [29] (PDB ID: 2GAU). The possible role of the N3A-motif in *trans*-regulated CNB domains needs to be elucidated, but the fact that the allosteric interface between α and β-subdomains is significantly different (Figure 7) indicates a possible difference in allosteric mechanisms.

The detection of four allosteric hot spots in PKA is consistent with NMR studies of the RIβ∶A-domain [16]. Our analysis shows that the N3A-motif contains two types of residues: hydrophobic X–X repeats, which provide structural stability to the element as well as its interactions within the CNB domain (Figure 3B and 3C). The other set of residues is positioned on the tip of the 3_10_-loop and is often involved in functionally important protein-protein interactions (Figure 3D).

The results of different *cis*-regulated CNB domains LSP-alignment were in good correspondence with our earlier analysis of CNB domain surface: all eleven residues that were found to be conserved [15], received very high IS values. Our results also support the proposed the “three shells” model of allostery [16]: high levels of IS were detected in the sites of all three shells localization: β_2_, β_3_ and β_8_ strands (Figure 4).

The most unexpected result was a very high scoring in the β_2,3_-loop region, which was not included previously in the general model of CNB domain allostery [14]. This brings up a question of the general role of the β_2,3_-loop in different CNB domains. As we showed, its residues always participate in multiple, highly conserved, polar and hydrophobic interactions with the PBC arginine (R^209^ Figure 6). The guanidinium group of this residue makes a very important hydrogen bond to the equatorial oxygen of the cAMP ribose phosphate. Perturbation of the bond, with an arginine-to-lysine mutation [30], or with substitution of the oxygen by sulfur [31], significantly disrupts the cAMP-related allostery. A close look at the β_2,3_-loop interactions with the PBC arginine, shows that it “surrounds” the residue, immobilizing its bulky side chain. The position and total geometry of the loop is remarkably conserved in all CNB domains. The central element of the loop is a universally conserved 3-turn between the positions #2 (D^164^) and #5 (D^167^). Spatial orientation of the turn is secured by another conserved interaction of residue #3 (Q^165^) with the β_4,5_-loop. The apparent reason for such rigidity is a correct positioning of the PBC arginine main chain, which is locked by two hydrogen bonds to residues #4 (G^166^) and #5 (D^167^). The tip of the arginine side chain is always bound to the #9 residue main chain (N^171^). A-domains of PKA regulatory subunits RIβ and RIIβ have an additional hydrogen bond to the side chain of residue #8 (D^170^). This interaction, however, is not conserved in other CNB domains. Alternatively, a polar bond between the eighth residue main chain and N-terminus of the B-helix was found in all studied structures. Thus, the possible role of the residue is to communicate between the PBC arginine and the hinge. The direct binding of residue #8 to the arginine, which is present in the PKA-R A-domains can substantially reinforce such communication.

**Figure 8:**
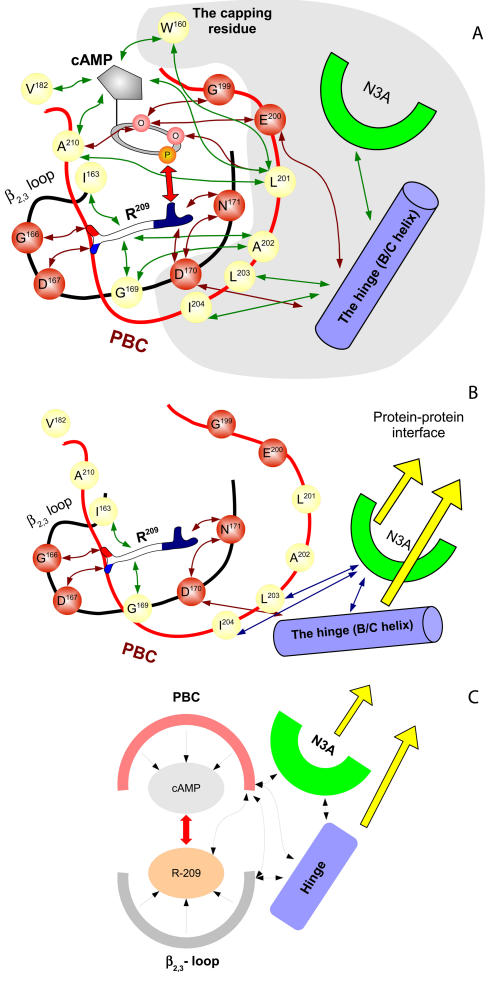
General allosteric mechanism for different CNB domains (RIα∶A case). (A) Major interactions between cAMP, the PBC (red) and the β_2,3_-loop (black) in cAMP-bound state. Red circles represent residues forming polar bonds (red arrows); yellow circles show residues making hydrophobic contacts (green arrows). The most important bond between cAMP and R^209^ is shown by a double red arrow. Residues and structure elements changing their positions upon cAMP binding are shaded grey. (B) cAMP-free configuration. R^209^ becomes much less restricted. (C) General diagram of major interactions in the CNB domain. The PBC controls cAMP, the 3_10_-loop controls R^209^, and their interaction provides correct orientation of the hinge region and the N3A motif, which form a protein-protein interface.

These finding lead us to a suggestion that immobilization of the PBC arginine side chain has to be an important feature of the allosteric mechanism. Definitely, without a stable guanidinium group, one hardly can expect a stable bond with the ribose phosphate moiety of cAMP. Here we propose a general model describing the cyclic nucleotide related allostery (Figure 8). The model is built around interactions between the cyclic nucleotide phosphate and the PBC-arginine guanidinium group (red arrow in Figure 8C). The main suggestion of the model is that the guanidinium group is very mobile and can not form a stable bond unless it is restricted by numerous interactions of the arginine with the PBC and β_2,3_-loop. This model is supported by the observation that the PBC-arginine side chain is not totally buried in the CNB domain, but partially exposed to the outer solution. According to the DSSP calculation [27], its water accessible area in the cAMP-free structures vary between 16 Å^2^ in the potassium channel to 30 Å^2^ in RIα∶A.

According to the proposed model stable cyclic nucleotide binding can be achieved only by interaction between all the major elements of the CNB domain: the PBC, the PBC-arginine, β_2,3_-loop, the hinge and the N3A-motif. Almost all elements interact with each other, leading to a rather complex allosteric mechanism. The primary function of the β_2,3_-loop is to position all parts of the arginine including the backbone, the hydrophobic part of the side chain and the guanidinium moiety. It also interacts with the hinge (through the residue #8) and the PBC (via hydrophobic contact between residue #7 and A^202^). The PBC serves, first of all, as a cyclic nucleotide molecule stabilizer, but it also plays an important role in immobilization of the arginine side chain (via A^202^). In addition, it interacts with the hinge and the N3A-motif, which usually forms protein-protein interface, providing the transition of the allosteric signal to the molecule, which contains the CNB domain.

### Conclusions

The LSP-alignment of H- and B-forms of different CNB domains revealed four conserved structural motifs: the PBC, the Hinge, the N3A-motif and the β_2,3_-loop. These elements were found in all studied *cis*-regulated CNB domains. The N3A motif is not present in CAP, which represents a *trans*-regulated CNB domain family. We propose a generalized allosteric mechanism for *cis*-regulated domains as follows: a) The PBC is a primary element, which binds sugar-phosphate moiety of cAMP. b) The β_2,3_-loop regulates the cAMP binding to the PBC via the conserved PBC-arginine. c) Both the PBC and the β_2,3_-loop communicate with the Hinge, which transfers the allosteric signal further to the N3A motif. d) The N3A-motif is the most malleable element of a CNB domain as it provides communication to the host protein.

## Methods

The following structures were used in the current work: PKA∶RIα B-form [32] (PDBID – 1RGS); PKA∶RIα H-form [8]; PKA∶RIIα H-form [20]; PKA∶RIIβ B-form [13] (1CX4); PKA∶RIIβ H-form (Brown et al., unpublished results); HCN B-form [33] (1Q43); MloK1 B-form [19] (1VP6); MloK1 H-form [19] (1U12); Epac2 H-form [34] (2BYV). LSP-alignment was made by previously reported algorithm for surface matching [21]. All residues (both water accessible and buried inside protein) were included in the analysis. For that reason, the water accessibility cut-off was taken equal to zero. Residues were represented by their C_α_–Cβ vectors. The maximum distance between C_α_ atoms was 12 Å. Tolerance for C_α_–C_α_ distance was 0.4 Å. Tolerance for C_α_–Cβ distance was 0.75 Å. Tolerance for the dihedral angle between the vectors was 30°. Residues with the BLOSUM62 score greater than or equal to 1 were considered to be similar. Calculations were made on a personal computer (Pentium 4; 1.8 GHz; 1 Gb RAM) under Linux OS. Molecular graphics were prepared using PyMOL (DeLano Scientific, San Carlos, CA).

## Supporting Information

Figure S1 Study of the cAMP-induced conformational changes. Changes in (A) PKA∶RIIβ (A-domain) and (B) potassium channel (MloK1) by LSP-alignment.
(0.09 MB DOC)
