# Correction: A Generalized Allosteric Mechanism for *cis*-Regulated Cyclic Nucleotide Binding Domains

**DOI:** 10.1371/annotation/43fd4ca3-0996-462c-ad89-395c369cbaa2

**Published:** 2009-07-01

**Authors:** Alexandr P. Kornev, Susan S. Taylor, Lynn F. Ten Eyck

In the Funding section, the number of the US National Institutes of Health grant to SST was listed incorrectly. The correct number is: GM34921.

