# Supplementary material for: A Generalized Allosteric Mechanism for cis-Regulated Cyclic Nucleotide Binding Domains
Source: PLoS Comput Biol. 2008 Apr 11;4(4):e1000056. doi: 10.1371/journal.pcbi.1000056 (PMC2275311; doi:10.1371/journal.pcbi.1000056)
Supplement: Figure S1 — Study of the cAMP-induced conformational changes. Changes in (A) PKA∶RIIβ (A-domain) and (B) potassium channel (MloK1) by LSP-alignment. (0.09 MB DOC) [file pcbi.1000056.s001.doc]

**Figure S1. Study of the cAMP induced conformational changes in a) PKA:RIIβ (A-domain) and b) potassium channel (MloK1) by LSP-alignment.** High involvement scores correspond to relatively rigid parts of the molecule. Low values of the score characterize elements, which are the most sensitive to the presence of cAMP. Secondary structure is shown by red rectangles (α-helix), magenta rectangles (3_10_-helix) and yellow arrows (β-strands). Four allosteric “hot spots” for each domain are shown by arrows.


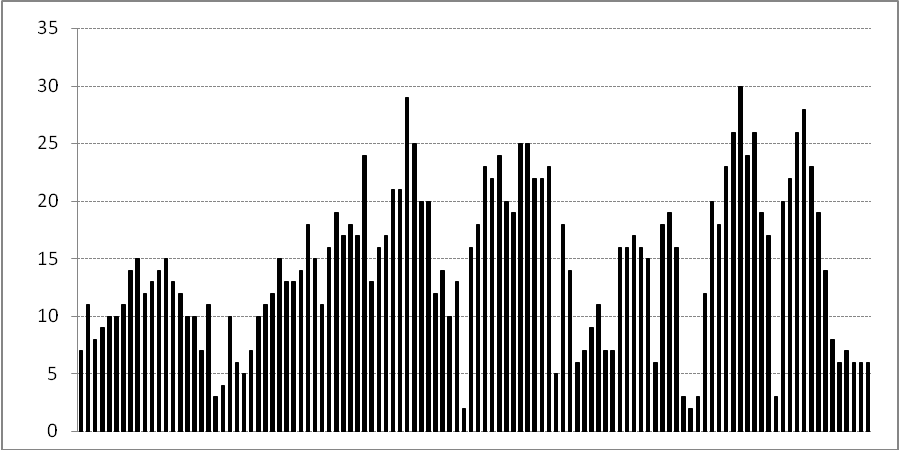

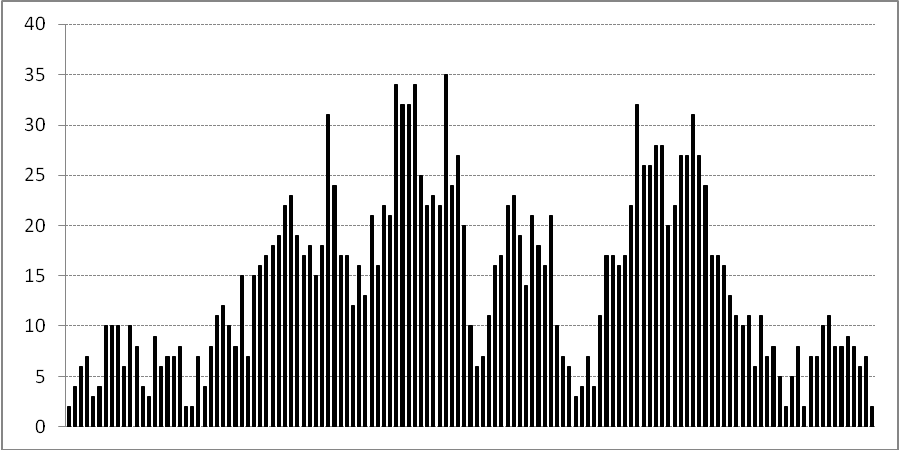


A

β1

β2

β3

β4

β5

β6

β7

β8

B

B

A

β1

β2

β3

β4

β5

β6

β7

β8

B’

B’

C

A

B
